# CMTM3 regulates vascular endothelial cell dysfunction by influencing pulmonary vascular endothelial permeability and inflammation in ARDS

**DOI:** 10.3389/fimmu.2025.1544610

**Published:** 2025-03-24

**Authors:** Ziyan Xiao, Gang Zhou, Haiyan Xue, Lihe Chen, Xiujuan Zhao, Shu Li, Chun Fu, Zhengzhou Wang, Fengxue Zhu

**Affiliations:** ^1^ Department of Critical Care Medicine, Peking University People’s Hospital, Beijing, China; ^2^ National Center for Trauma Medicine of China, Peking University People's Hospital, Beijing, China; ^3^ Beijing Key Surgical Basic Research Laboratory of Liver Cirrhosis and Liver Cancer, Peking University People’s Hospital, Beijing, China

**Keywords:** ARDS, HUVECs, CMTM3, inflammation, immunosuppression

## Abstract

**Introduction:**

CMTM3 is a member of the human chemokine-like factor superfamily. The mechanistic role of CMTM3 in acute respiratory distress syndrome (ARDS) is not known. This study investigated the role of CMTM3 in the progression of ARDS and its impact on the function of vascular endothelial cells.

**Methods:**

ARDS modeling in human umbilical vascular endothelial cells (HUVECs) was performed by treating with lipopolysaccharide (LPS) or hypoxia/reoxygenation. We assessed CMTM3 expression levels in the LPS- and hypoxia/reoxygenation-stimulated HUVEC cells. Furthermore, we assessed the role of CMTM3 in the permeability function and inflammatory response of the vascular endothelial cells under ARDS conditions using HUVEC cells with CMTM3 overexpression(adCMTM3) or knockdown(shCMTM3). Concurrently, we generated CMTM3 knockout (CMTM3ko) mice and evaluated the differences in pulmonary vascular permeability, inflammatory lung injury, and survival rates between the CMTM3ko-ARDS and WT-ARDS model mice.

**Results:**

HUVECs stimulated with LPS and hypoxia/reoxygenation showed significantly higher CMTM3 expression compared to the control group (p<0.05). Compared with the adsham-HUVECs, adCMTM3-HUVECs stimulated with LPS and hypoxia/reoxygenation demonstrated significantly higher cellular permeability (p<0.05) as well as IL-6 and TNF-α expression levels (p<0.05). Conversely, shCMTM3-HUVECs stimulated with LPS and hypoxia/reoxygenation showed significantly reduced cellular permeability as well as IL-6 and TNF-α expression levels (p<0.05). In vivo ARDS modeling experiments demonstrated that CMTM3-knockout ARDS mice exhibited significantly higher survival rates (p=0.0194) as well as significantly reduced lung injury and pulmonary vascular permeability (p<0.05) compared to the wild-type ARDS mice.

**Discussion:**

These findings demonstrated that CMTM3 played a critical role in the development of ARDS by influencing permeability of the pulmonary vascular endothelial cells and lung inflammation. Therefore, CMTM3 is a potential therapeutic target in ARDS.

## 1 Introduction

Acute Respiratory Distress Syndrome (ARDS) is characterized by progressive hypoxemia and respiratory distress (1). The pathophysiology of ARDS involves damage to the pulmonary capillary endothelial cells and alveolar epithelial cells. This leads to increased permeability of the alveolar membranes, destruction of the alveolar surfactants, formation of hyaline membranes, and alveolar atrophy. This causes diffuse interstitial and alveolar edema in the course of non-cardiogenic illnesses such as severe infections, trauma, and shock. ARDS is one of the most common and serious complications during sepsis (2, 3). About 25% to 50% of patients with severe infections develop ARDS (4). The incidence of ARDS increases with the severity of sepsis. The mortality rate of patients with sepsis-related ARDS is 70% to 90% (5).

Barrier dysfunction is the primary mechanism underlying ARDS and is caused by inflammatory mediators and cytokines (6) as well as oxidative stress. The process of ARDS mainly involves dysregulated inflammatory response and excessive production of cytokines, multinucleated cells, monocytes, macrophages, vascular endothelial cells, and the coagulation and fibrinolytic system. In ARDS, acute inflammatory response directly damages the pulmonary capillary endothelial cells and leads to a cascade of events that indirectly damages the lung tissue (7). Pulmonary capillary endothelial cells represent 20%–30% of the total cell population in the lungs. They play an important role in regulating pulmonary vascular tone and maintaining the function and integrity of the alveolar-pulmonary capillary membrane barrier (7). Pulmonary capillary endothelial cells are both the target cells and effector cells in the ARDS-related inflammatory response. Endothelial barrier dysfunction caused by damage to the lung endothelial cells leads to increased permeability of the vascular endothelium to the plasma proteins and inflammatory cells. This results in pulmonary edema, which leads to ventilation dysfunction and the development of ARDS (8). Currently, clinically approved drugs that effectively target the inflammatory response or endothelial damage are not available for patients with ARDS. Therefore, there is an urgent need to characterize the pathogenetic mechanism underlying endothelial dysfunction in ARDS to identify new targets for the treatment of endothelial barrier disruption and vascular inflammation to slow down disease progression during early stages of ARDS and improve the prognosis of patients.

The CKLF-like MARVEL transmembrane structural domain-containing family (CMTM) is a family of genes with immune functions. It consists of nine genes, including chemokine-like factor (CKLF) and CMTM1-8, which share significant protein sequence homology (9, 10).

CMTM3, a key member of the CMTM family, contains a MARVEL structural domain, which regulates membrane penetration and protein secretion (11). CMTM3 is highly conserved during evolution. The human and murine forms of CMTM3 show 91.8% protein sequence homology (9). CMTM3 plays contradictory roles in different tumors. It functions as an oncogene in gastric cancer and hepatocellular carcinoma (12, 13) but plays a pro-carcinogenic role in gliomas (14). This suggests tissue and cellular heterogeneity in the expression and function of CMTM3. Furthermore, CMTM3 mediates angiogenesis by regulating the levels of VE-cadherin on the surface of endothelial cells at the adherens junction cells, thereby impacting vascular endothelial cell permeability (15). Vascular endothelial function plays a significant role in the pathogenesis of ARDS, but the role and mechanism of CMTM3 in ARDS are not known. Therefore, in this study, we investigated whether CMTM3 played a role in ARDS by affecting vascular endothelial function. Our aim was to provide a scientific basis for CMTM3 as a novel diagnostic biomarker and therapeutic target in ARDS.

## 2 Materials and methods

### 2.1 Mice

We obtained 6- to 8-week-old CMTM3 knockout mice and wild-type (WT) mice with the same genetic background from the laboratory of Prof. Wenling Han, Department of Immunology, Peking University School of Medicine. These mice underwent a controlled breeding program to ensure genetic uniformity. All the mice were housed under specific pathogen-free (SPF) conditions at the Animal Center of the People’s Hospital of Peking University (**Table 1**). The environmental conditions were strictly controlled with a temperature range of 22°C–25°C, humidity of 50%–60%, and a 12-h light-dark cycle. The mice were provided free access to food and water. For the experiments, we selected 8- to 10-week-old mice weighing 18–20 g. The mouse experiments adhered to all ethical and moral guidelines for animal experimentation. The experimental protocol was reviewed and approved by the Animal Experimentation Ethics Committee of the People’s Hospital of Peking University (Approval 465 No. 2022PHE058).

**Table 1:** Mice, cell lines, and reagents.

Reagent/resource	Reference or source	Identifier or catalog number
Experimental models		
Cmtm3^-/-^ C57/BL6 mice	Professor Han Wenling’s laboratory at Peking University Health Science Center	N/A
Cmtm3^+/+^ C57/BL6 mice	Professor Han Wenling’s laboratory at Peking University Health Science Center	N/A
Experimental cells		
HUVECs	Meisen (CTCC)	N/A
Chemicals, Enzymes and other reagents		
Endothelial Cell Medium	ScienCell	1001
CMTM3 overexpression Lentivirus	Shanghai Jikai Gene Chemistry Co. China	N/A
CMTM3 knockdown Adenovirus	Shanghai Jikai Gene Chemistry Co. China	N/A
Puromycin	Solarbio	P8230
25cm² Rectangular Canted Neck Cell Culture Flask with Vent Cap	Corning	430639
Lipopolysaccharide (MICE)	Solarbio	L8880
Lipopolysaccharide (HUVECs)	Solarbio	IL2020
Evans Blue Stain Solution 0.5%	Solarbio	G1810
HCL (0.1mol/L)	Godow	N/A
TRIzol^®^ Reagent	InvitrogenTM	15596026
RevertAid First Strand cDNA Synthesis Kit	Thermo Scientific	K1622
SYBR^®^ Green real-time PCR master mix	TOYOBO	QPK-201
FITC-Dextran (MW 10000)	MedChemExpress (MCE)	HY-128868
Transwell Permeable Support with 0.4µm Pore Polyester (PET) Membrane	Corning	3470
AnaeroPack-Anaero5%	MITSUBISHI	C-04
2.5L anaerobic culture tank/culture box	MITSUBISHI	C-31
Oxygen Indicator	MITSUBISHI	C-22
Formamide	LABLEAD	0314

### 2.2 Generation of the ARDS model mice using the HCL/LPS tracheal drip

After inducing general anesthesia and analgesia, the experimental group mice were administered 0.1 ml of HCL (pH = 1.0)/0.1 ml of LPS (10 mg/ml), and the control group mice were administered 0.1 ml of saline through the tracheal drip. After the operation, the mice were carefully transferred to the recovery cages and rewarmed to gain full recovery and consciousness as previously described (16, 17).

### 2.3 Determination of lung permeability in mice using the Evans blue dye

We first generated the standard curve of the EBD/formamide solution. The lung samples were harvested at 24h after ARDS modeling. Evans blue dye (0.1ml/0.5%) was injected intravenously into the mice 15 min before sampling. The lungs were removed from the chest, and the surface water was dried with filter paper. After removing the surrounding tissues, the lung tissues were cut into smaller pieces, immersed in formamide solution (100 g/20 ml), and incubated at 37°C for 72h in an incubator. After centrifugation, the supernatant was analyzed using a fluorescence spectrophotometer. The amount of Evans blue dye per gram of lung tissue was calculated using the Evans blue-formamide standard curve and reflected pulmonary vascular permeability.

### 2.4 H&E staining of mouse lung tissue sections

The mice were euthanized after 24h of ARDS modeling. The lung tissue was removed and washed with PBS solution to remove blood. Subsequently, part of the lung tissues were incubated in 4% paraformaldehyde at 4°C overnight. The next day, the tissue samples were dehydrated in 70%–100% ethanol and embedded in paraffin. The paraffin-embedded lung tissues were then sectioned. Subsequently, deparaffinized sections (5 μm thick) were stained with hematoxylin and eosin (H&E) according to standard procedures described previously and photographed under a microscope to observe morphological characteristics, and the Smith scoring method was used to quantify lung injury (Take 10 visual fields and get the average score: Semi-quantitative Analysis of Pulmonary Edema, Alveolar and Interstitial Inflammation, Alveolar and Interstitial Hemorrhage, Atelectasis, and Hyaline Membrane Formation (0–4 Points):0 Points: No injury; 1 Point: Lesion area <25%; 2 Points: Lesion area 25%–50%; 3 Points: Lesion area 50%–75%; 4 Points: Lesion area occupying the entire field of view).

### 2.5 HUVEC culturing

Human umbilical vein endothelial cells (HUVECs) were cultured in the ECM (ScienCell, USA) medium at 37°C and 5% CO_2_ in a humidified incubator. The cells were regularly assessed for mycoplasma contamination. Cells were passaged twice a week. They were washed with FBS, digested with trypsin (0.05%) solution, and resuspended in ECM medium.

### 2.6 Generation of HUVECs with stable CMTM3 overexpression

The CMTM3 overexpression lentiviral vector was constructed by the Shanghai Jikai Gene Chemistry Co. Ltd (Shanghai, China). We constructed plasmids carrying the target gene as well as helper plasmids for viral packaging; these plasmids were co-transfected into HEK293T cells using the liposome method. The viral seeds were subsequently harvested and amplified, followed by purification of the virus. Through this process, we successfully generated adenoviruses overexpressing CMTM3 (adCMTM3) and a control adenovirus (with sham virus containing an empty expression vector, adsham). HUVECs were transduced with different concentrations of the adenoviruses particles encoding the *CMTM3* gene to screen for appropriate MOI values (MOI = 10). The transduced cells were screened with ECM medium containing 1 μg/ml puromycin to identify stably transduced strains, which were then used for subsequent experiments.

### 2.7 Generation of HUVECs with CMTM3 knockdown

To target the CMTM3 gene sequence, shRNA sequences were designed according to RNA interference (RNAi) principles for the construction of shRNA plasmids. The lentiviral backbone used in this study contains a puromycin resistance gene for eukaryotic selection. After obtaining the purified plasmid with confirmed correct sequencing, lentivirus packaging will be carried out using HEK293T cells. Subsequently, HUVECs were co-cultured with different concentrations of lentivirus containing CMTM3 shRNA and a control lentivirus (with sham virus containing an empty expression vector, shsham) after being screened for appropriate MOI values (MOI = 10). The selection of stable transfectants via puromycin resistance and the transduced cells were passaged in the ECM medium for further experiments.

### 2.8 LPS-stimulated/hypoxia/reoxygenation-stimulated HUVEC models

HUVECs growing exponentially were incubated with different concentrations of LPS, and total cellular RNA was extracted at different time points and analyzed by real-time quantitative reverse transcription polymerase chain reaction (qRT-PCR). After initial screening, subsequent experiments were performed for 6h using 100 ng/ml LPS. Exponentially growing HUVECs were placed in a hypoxia tank (for 4h or 5h) with an AnaeroPack-Anaero5% (to ensure 1% O_2_ and 5% CO_2_). The cells were reoxygenated for 2h after hypoxic exposure for different time periods (place the hypoxia tank in a normal incubator to ensure the temperature and humidity required for cell growth). Total cellular RNA was extracted from the cells and analyzed by qRT-PCR. After initial screening, the cells were exposed to 4h of hypoxia and 2h of reoxygenation for the subsequent experiments.

### 2.9 Real-time fluorescence quantitative PCR

Total cellular RNA was extracted using the Trizol method and quantified. Subsequently, cDNA was prepared by reverse transcription using the Revertra Ace qPCR RT Master Mix with gDNA Remover according to the manufacturer’s instructions. Then, qPCR was performed using the SYBR^®^ Green Realtime PCR Master Mix. The expression levels of the target genes were estimated relative to GAPDH using the double delta Ct value method (2^-ΔΔCt^). The qPCR primers are listed in **Table 2**.

**Table 2:** Quantitative PCR primers.

Gene name	Direction	Primer sequence (5′ to 3′)
Human qPCR primer pair		
CMTM3	Forward	AATGACAAGTGGCAGGGCT
	Reverse	TTGTGGGCTGTGGTCTCAT
IL-6	Forward	AGACAGCCACTCACCTCTTCAG
	Reverse	TTCTGCCAGTGCCTCTTTGCTG
TNF-α	Forward	CTCTTCTGCCTGCTGCACTTTG
	Reverse	ATGGGCTACAGGCTTGTCACTC
GAPDH	Forward	GAAGGTGAAGGTCGGAGTC
	Reverse	GGAAGATGGTGATGGGATTT
Mouse qPCR primer pair		
IL-6	Forward	TACCACTTCACAAGTCGGAGGC
	Reverse	CTGCAAGTGCATCATCGTTGTTC
TNF-α	Forward	GGTGCCTATGTCTCAGCCTCTT
	Reverse	GCCATAGAACTGATGAGAGGGAG
GAPDH	Forward	CATCACTGCCACCCAGAAGACTG
	Reverse	ATGCCAGTGAGCTTCCCGTTCAG

### 2.10 Estimation of cell permeability using the FITC-dextran assay

The cells were trypsin-digested, harvested, and resuspended in ECM medium. Then, 200 µl (1 × 10^5^) cells were seeded in the upper chamber of the Transwell, 600 µl ECM medium in the down chamber, and cultured cells attached and reached a monolayer cell barrier layer. Then, after LPS-stimulated/hypoxia/reoxygenation-stimulated, 0.1 mg/ml FITC (fluorescein isothiocyanate)-labeled dextran was added to the ECM medium in the upper chamber of the transwell. After 15 min of incubation, medium from the lower chamber of the transwell was collected and the fluorescence signals were estimated using the Fluoroskan Ascent FL plate reader (Thermo Scientific, Waltham, MA) at 485 nm excitation and 538 nm emission. Comparative FITC-dextran concentrations were calculated based on fluorescence intensity to assess the cell layer permeability.

### 2.11 Transcriptome sequencing

Total RNA was isolated from the CMTM3-knockdown cells using the Trizol reagent. Subsequently, mRNA sample libraries were constructed, and high-throughput sequencing was performed by the Shanghai Zhongke New Life Biotechnology Co. Ltd. (Shanghai, China). Briefly, after quantitation of the total RNA samples, the mRNA was purified by poly-dT oligo-linked magnetic beads. The enriched mRNA fragments were then divided into shorter fragments. The fragmented mRNAs were used as templates for library construction. Once the libraries passed quality control, the sequencing data was generated using the Illumina/BGI platform, and bioinformatics analysis (GOrich, KEGG, PPI) was performed.

### 2.12 Statistical analysis

Statistical analysis was performed using the GraphPad Prism 8.4.1 software (GraphPad Software Inc., San Diego, CA, USA). Unpaired two-tailed Student’s t-test was used for comparisons between two groups. One-way ANOVA and Dunnett’s *post hoc* test were used for comparisons between more than two groups. *P* < 0.05 was considered statistically significant.

## 3 Results

### 3.1 CMTM3 expression levels are significantly altered in the LPS or hypoxia/reoxygenation-stimulated HUVECs

**Figure 1:**
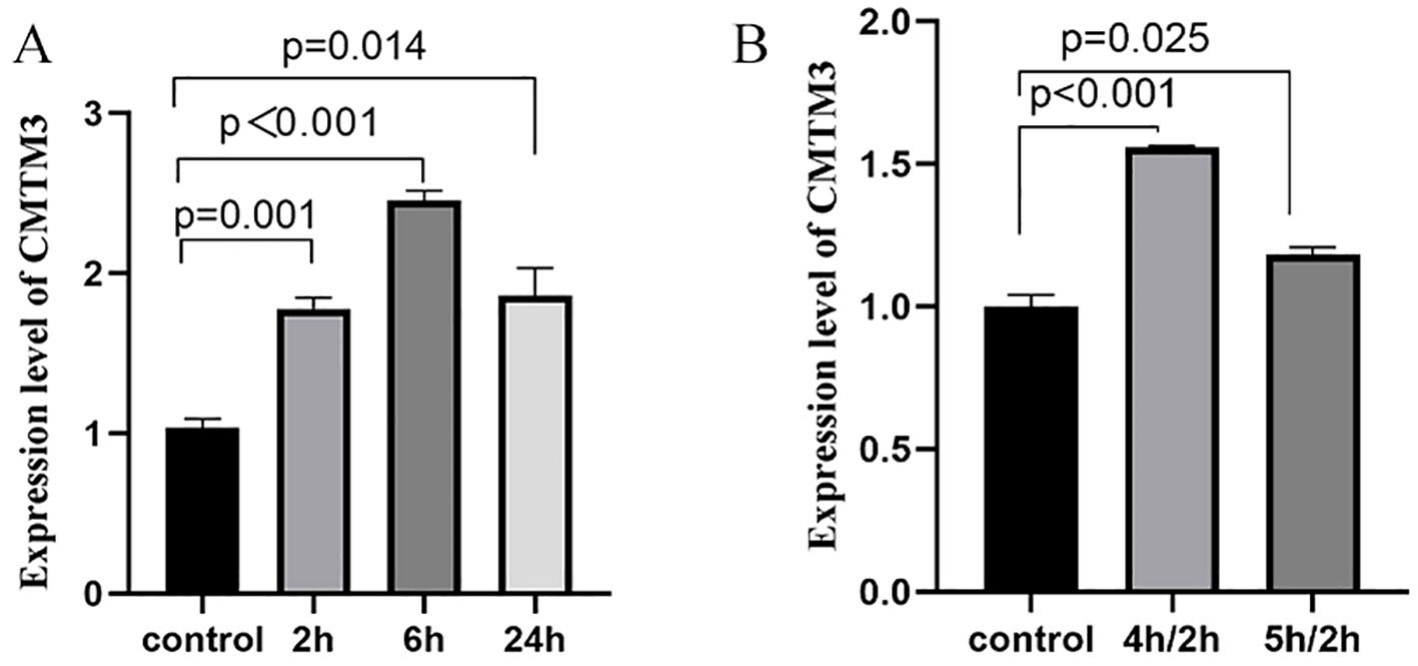
**(A)** The expression levels of CMTM3 transcripts in human umbilical vein endothelial cells (HUVECs) treated with 100 ng/ml LPS for 2h, 6h, and 24h (*n* ≧ 3). **(B)** The expression levels of CMTM3 transcripts in HUVECs subjected to stimulation with 4h-hypoxia/2h-reoxygenation and 5h-hypoxia/2h-reoxygenation. Unstimulated HUVECs were used as the control (*n* ≧ 3).

Initially, we generated *in-vitro* ARDS models in the HUVECs and analyzed CMTM3 expression levels under ARDS inflammatory conditions. To simulate ARDS *in vitro*, we used lipopolysaccharide (LPS) stimulation or hypoxia followed by reoxygenation (hypoxia/reoxygenation). First, we stimulated exponentially growing HUVECs with100 ng/ml of LPS for 2h, 6h, and 24h, and analyzed CMTM3 levels. CMTM3 expression levels were significantly higher in the LPS-stimulated HUVEC cells at all time points compared to the control unstimulated HUVECs, with 6h time point showing the highest level of CMTM3 expression (*p* < 0.001) (**Figure 1A**). In the hypoxia/reoxygenation ARDS model, HUVEC cells were subjected to hypoxia for 4h followed by reoxygenation for 2h or hypoxia for 5h followed by reoxygenation for 2h Compared with the unstimulated HUVECs, the expression levels of CMTM3 were significantly increased by hypoxia/reoxygenation treatment and were highest in the 4h-hypoxia/2h-reoxygenation-treated HUVECs (*P* < 0.001) (**Figure 1B**).

### 3.2 Changes in the permeability of HUVECs with CMTM3 overexpression or knockdown after LPS or hypoxia/reoxygenation stimulation

**Figure 2:**
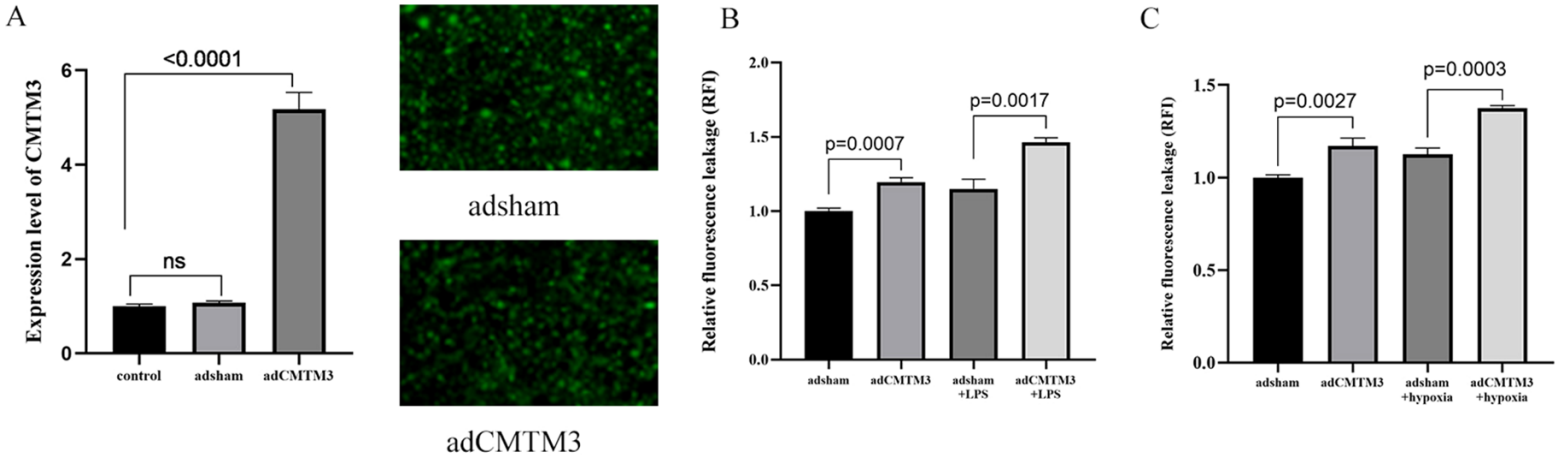
CMTM3 overexpression enhances permeability of the human umbilical vein endothelial cells (HUVECs) under *in-vitro* ADRS inflammatory conditions (*n* ≧ 3). **(A)** CMTM3 expression levels in control HUVECs as well as adsham and adCMTM3-transfected HUVECs. **(B)** FITC-dextran assay results show the cellular permeability based on relative fluorescence leakage (RFI) in the adsham- and adCMTM3-transfected HUVECs that were untreated or treated with 100 ng/ml LPS for 6h. **(C)** FITC-Dextran assay results show the cellular permeability based on relative fluorescence leakage (RFI) in the adsham- and adCMTM3-transfected HUVECs that were untreated or treated with 4h-hypoxia/2h-reoxygenation.

To investigate the effect of CMTM3 overexpression on the vascular endothelial cell permeability during ARDS-induced inflammatory state, we transfected HUVECs with lentiviruses carrying the CMTM3-overexpressing vector at a MOI of 10 and screened for stably transfected HUVECs overexpressing CMTM3 (adCMTM3) (*p* < 0.0001) (**Figure 2A**). Subsequently, we analyzed permeability of the control(adsham) and adCMTM3 HUVECs in the monolayer using the FITC-Dextran assay, adCMTM3HUVECs showed significantly higher endothelial cell permeability (RFI) under unstimulated conditions (*p* = 0.0007, *p* = 0.0027) (**Figures 2B, C**), as well as when stimulated with 100 ng/ml LPS for 6h (*P* = 0.0017) (**Figure 2B**) and after 4h hypoxia and 2h of reoxygenation (*p* = 0.003) (**Figure 2C**) compared with the corresponding controls.

**Figure 3:**
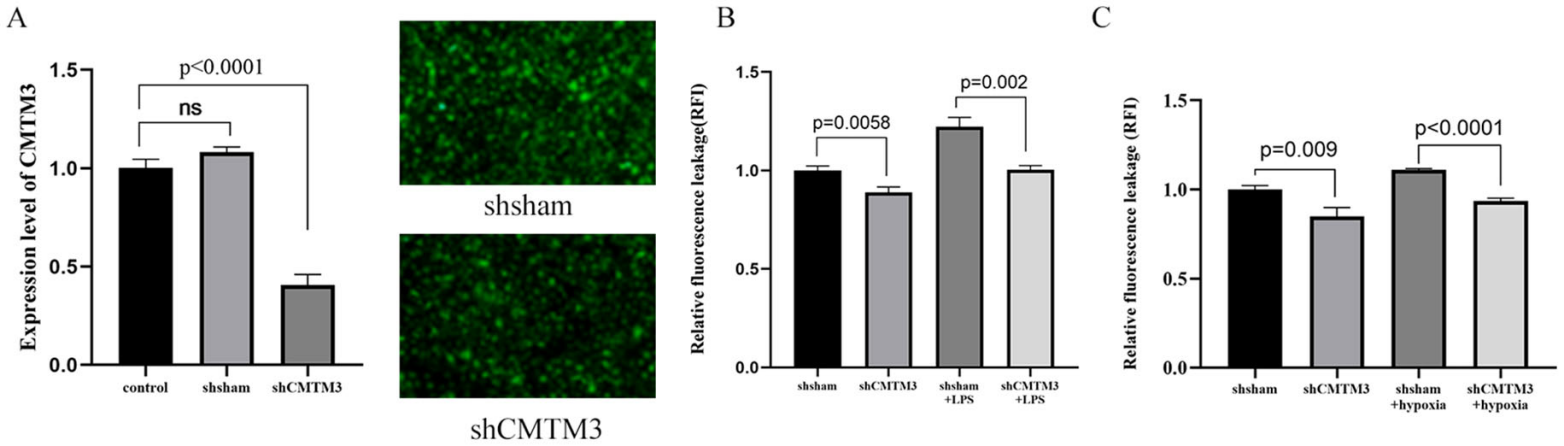
CMTM3 knockdown reduces human umbilical vein endothelial cell (HUVEC) permeability under *in-vitro* inflammatory conditions. **(A)** CMTM3 expression levels in the control, shsham, and shCMTM3 HUVECs. **(B)** FITC-dextran assay results show the cellular permeability based on relative fluorescence leakage (RFI) in the shsham and shCMTM3-transfected-HUVECs that were untreated or treated with 100 ng/ml LPS for 6h. **(C)** FITC-dextran assay results show the cellular permeability based on relative fluorescence leakage (RFI) of shsham and shCMTM3-transfected HUVECs that were untreated or treated with 4h-hypoxia/2h-reoxygenation.

Next, to further investigate the role of CMTM3 in vascular endothelial cell permeability, we transfected HUVECs with lentivirus containing CMTM3 shRNA at a MOI of 10 to knockdown CMTM3 expression levels in the HUVECs and verified the CMTM3 knockdown (shCMTM3-HUVECs) levels against shsham HUVECs (*p* < 0.0001) (**Figure 3A**). Subsequently, we compared the cell permeability of the control and shCMTM3 HUVECs in the monolayer. FITC-Dextran assay results demonstrated that the permeability of shCMTM3 HUVECs was significantly reduced compared with the shsham HUVECs (*p* = 0.0058, *p* = 0.009) (**Figure 3B**). Furthermore, when stimulated with 100 ng/ml LPS for 6h, cellular permeability of the shCMTM3-HUVECs was significantly lower than the control group (*P* = 0.002) (**Figure 3B**). Moreover, when treated with 4h of hypoxia followed by 2h of reoxygenation, cellular permeability of the shCMTM3-HUVECs was significantly lower than the shsham HUVECs (*P* < 0.0001) (**Figure 3C**).

### 3.3 Evans blue leakage assay demonstrates reduced lung leakage in the CMTM3 knockout ARDS model mice

**Figure 4:**
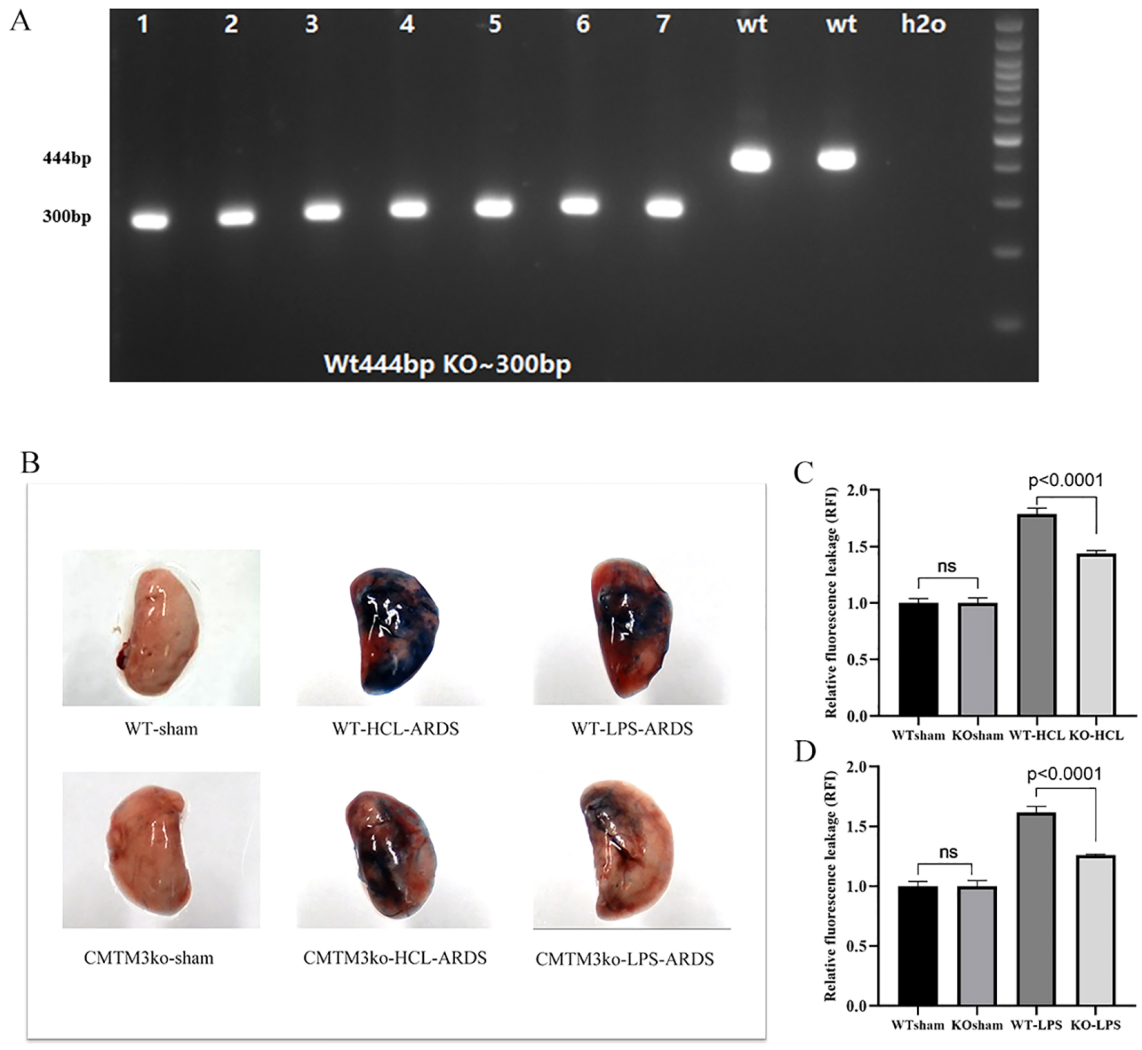
*In-vivo* analysis of lung tissue permeability in the CMTM knockout ARDS model mice (*n* = 5 per group). **(A)** Polymerase chain reaction (PCR) analysis confirming the genotypes of the in-bred CMTM3 knockout mice. CMTM knockout mice show a 300 bp PCR product compared to the 444 bp product in the wild-type (WT) mice. **(B)** Picture of Evans blue dye leakage analysis of lungs after HCl and lipopolysaccharide (LPS)–based acute respiratory distress syndrome (ARDS) modeling **(C)** Evans blue dye leakage analysis of lungs after HCl-based ARDS modeling of the WT and CMTM knockout mice. **(D)** Evans blue dye leakage analysis of lungs after LPS-based ARDS modeling of the WT and CMTM knockout mice.

To further confirm the *in-vivo* function of CMTM3 in the lung vascular endothelial permeability and ARDS, we generated CMTM3 knockout mice (**Figure 4A**).

In the HCL-ARDS model, we observed significantly lower leakage of Evans blue dye from the lungs of the CMTM3-KO mice compared to the WT mice as shown in the lung tissue images (**Figure 4B**). Quantification of Evans blue dye fluorescence also showed significantly reduced leakage from the lungs of the CMTM3KO-ARDS model mice compared with the WT-ARDS model mice (*P* < 0.0001, **Figure 4C**). Similar results were obtained in the LPS-ARDS model. The WT mice showed significantly higher extent of lung injury and EB dye leakage (*p* < 0.0001) (**Figure 4D**) compared to the CMTM3-KO mice after 24h of ARDS modeling. However, there were no significant differences in the EB dye leakage between the lungs of sham-treated WT and CMTM3-KO mice (*p* > 0.05).

### 3.4 Effect of CMTM3 on lung inflammation

**Figure 5:**
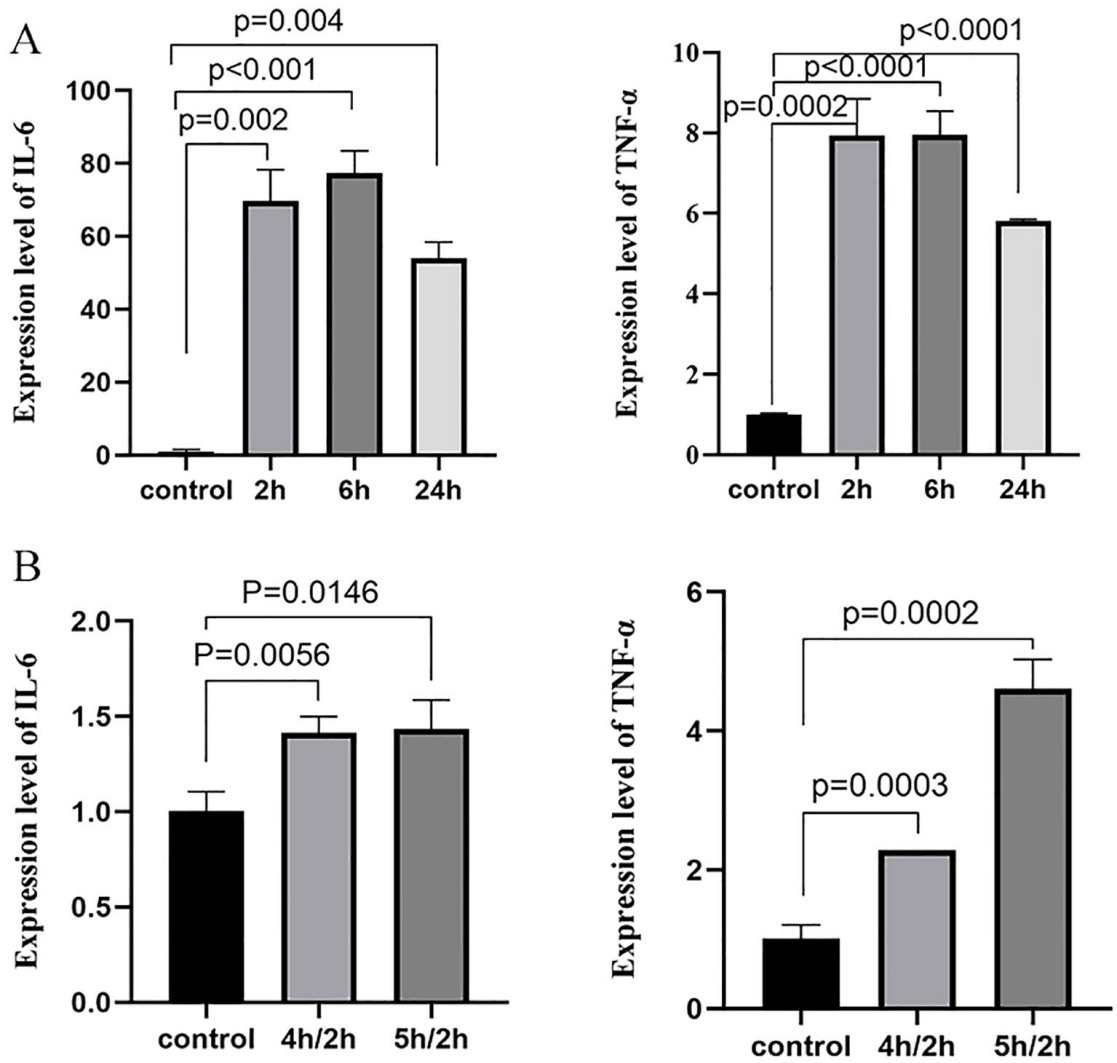
IL-6 and TNF-α expression levels after **(A)** stimulation with 100 ng/ml LPS for 2h, 6h, and 24h, and **(B)** stimulation with 4h/2h and 5h/2h hypoxia/reoxygenation. (*n* ≧ 3).

To investigate the effects of CMTM3 on the inflammatory state in ARDS, we first *in vitro* stimulated HUVECs with LPS for 2h, 6h, and 24h or with hypoxia (4h or 5h)/2h reoxygenation and analyzed the levels of inflammatory cytokines IL-6 and TNF-α. In the LPS model of ARDS, both IL-6 and TNF-α levels increased significantly and reached their peak at the 6h time point (*p* < 0.001 and *p* < 0.0001; **Figure 5A**). In the hypoxia/reoxygenation model of ARDS, the levels of IL-6 and TNF-α increased significantly compared with the control and reached peak levels when stimulated with hypoxia for 5h followed by 2h of reoxygenation (*p* = 0.0146 and *p* = 0.0002) (**Figure 5B**).

**Figure 6:**
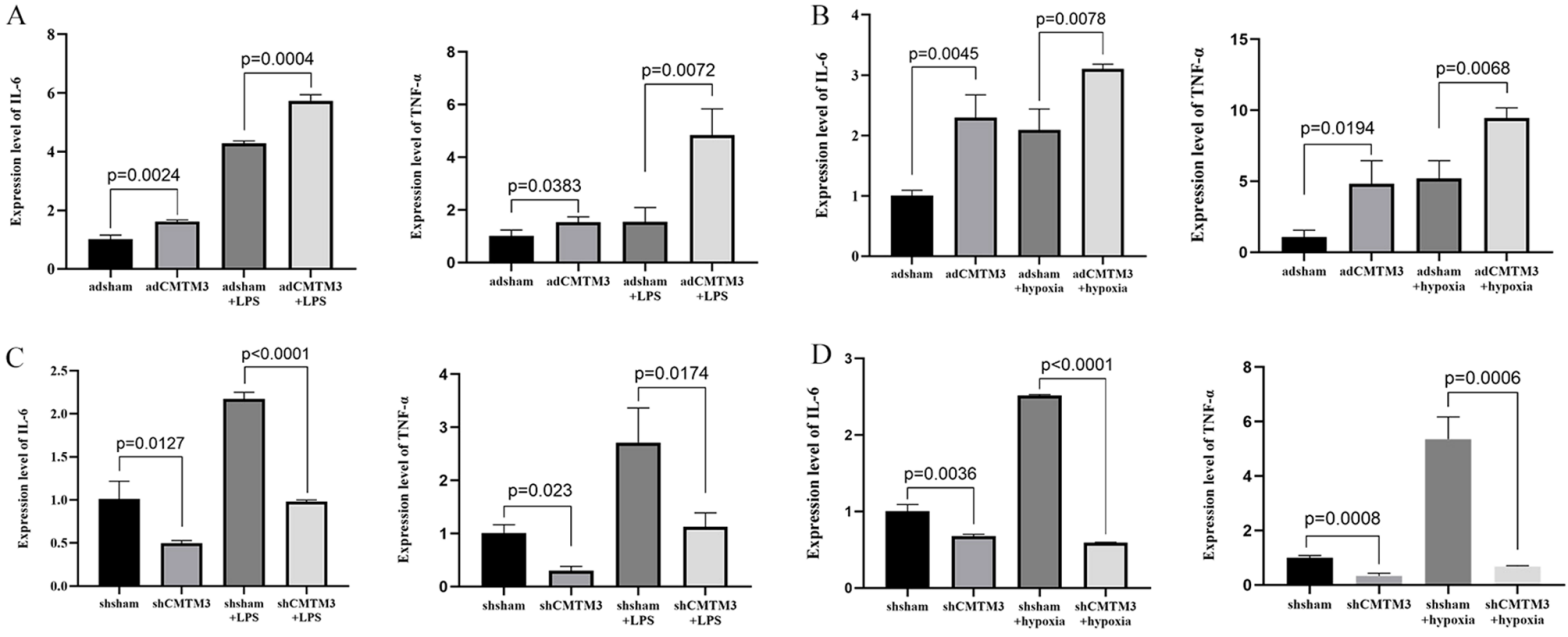
The expression levels of IL-6 and TNF-α in the adCMTM3- and shCMTM3-transfected HUVECs after **(A, C)** LPS stimulation for 6 h and **(B, D)** 4h/2h hypoxia/reoxygenation treatment.

Subsequently, CMTM3-overexpressing or knockdownHUVECs were treated with LPS to determine the potential effects of CMTM3 expression on the ARDS-related inflammatory state. Compared with the controls, IL-6 and TNF-α levels in the CMTM3-overexpressing HUVECs were significantly elevated when stimulated with LPS (*p* = 0.0004 and *p* = 0.0072; **Figure 6A**) and 4h/2h hypoxia/reoxygenation (*p* < 0.0001 and *p* = 0.0174; **Figure 6B**). In contrast, CMTM3-knockdown HUVECs demonstrated significantly reduced levels of IL-6 and TNF-α after stimulation with LPS (*p* = 0.0078 and *p* = 0.0068; **Figure 6C**) and 4h/2h hypoxia/reoxygenation (*P* < 0.0001 and *p* = 0.0006; **Figure 6D**).

### 3.5 Effect of CMTM3 knockout on the lung inflammatory status in the ARDS model mice

**Figure 7:**
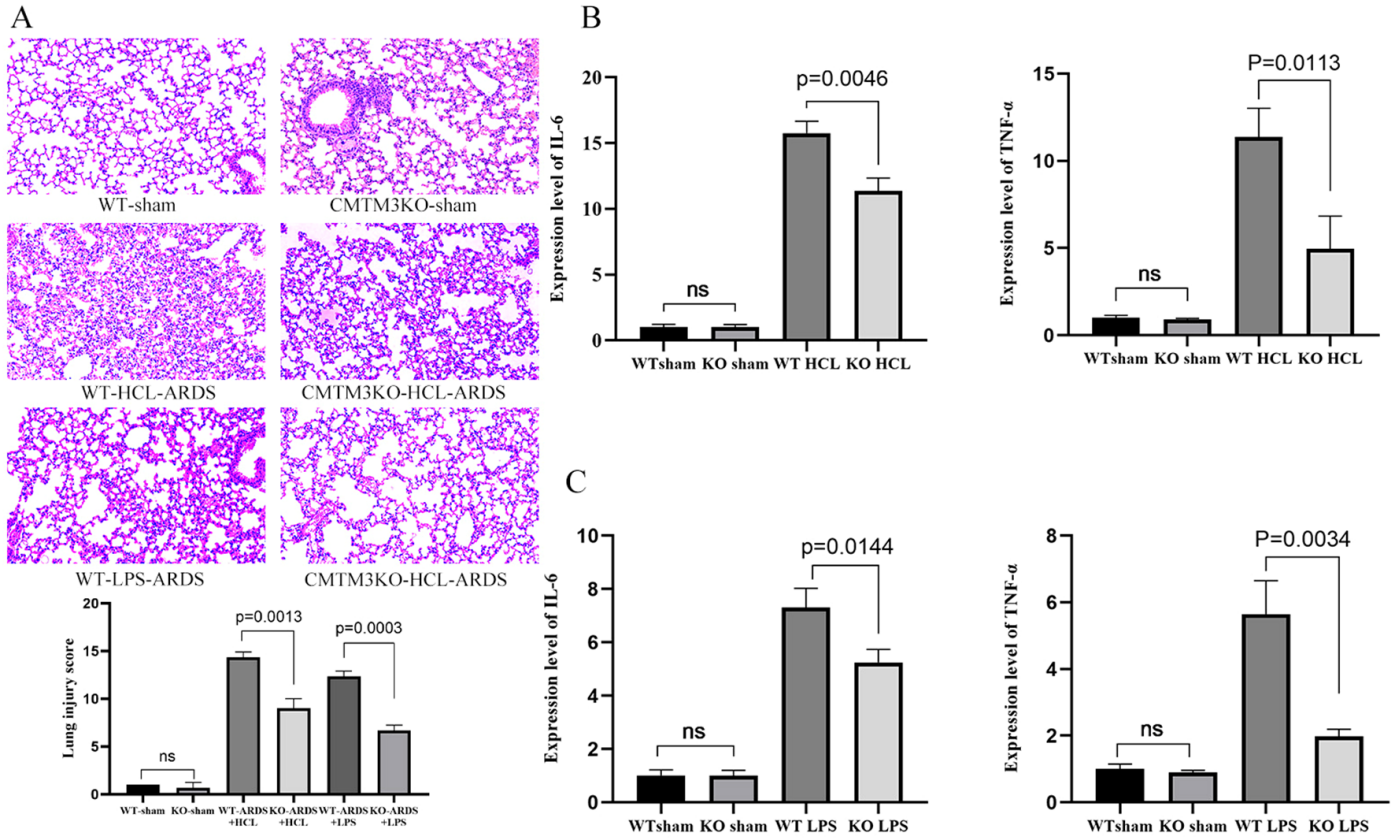
**(A)** Pictures and Smith scoring of lung injury of wild-type (WT) and CMTM3ko mice after tracheal drip dilute HCL/lipopolysaccharide (LPS) modeling (*n* = 5 per group). **(B)** Lung injury and expression levels of IL-6 and TNF-α in the lungs of the WT and CMTMko mice after tracheal drip dilute HCL modeling (*n* = 5 per group). **(C)** Lung injury and expression levels of IL-6 and TNF-α in the lungs of the WT and CMTMko mice after tracheal drip LPS modeling (*n* = 5 per group).

Next, we analyzed the degree of lung injury and inflammation status in the ARDS model CMTM3KO and WT mice. ARDS modeling was performed by two different methods, namely, tracheal drip dilute HCL (WT-HCL-ARDS and CMTM3KO-HCL-ARDS mice) and tracheal drip LPS (WT-LPS-ARDS and CMTM3KO-LPS-ARDS mice). The expression levels of inflammatory factors and lung injury were analyzed in the mice after 24h of modeling. CMTM3KO-HCL-ARDS mice showed reduced lung injury (**Figure 7A**, p = 0.0017), and significantly lower expression levels of IL-6 and TNF-α (*p* = 0.0046 and *p* = 0.0113; **Figure 7B**) than the WT-HCL-ARDS mice. Furthermore, CMTM3KO-LPS-ARDS mice showed reduced lung injury (**Figure 7A**, p = 0.0003) and significantly lower expression levels of IL-6 and TNF-α (*p* = 0.0144 and *p* = 0.0034; **Figure 7C**) compared to the WT-LPS-ARDS mice.

### 3.6 RNA sequencing data analysis

**Figure 8:**
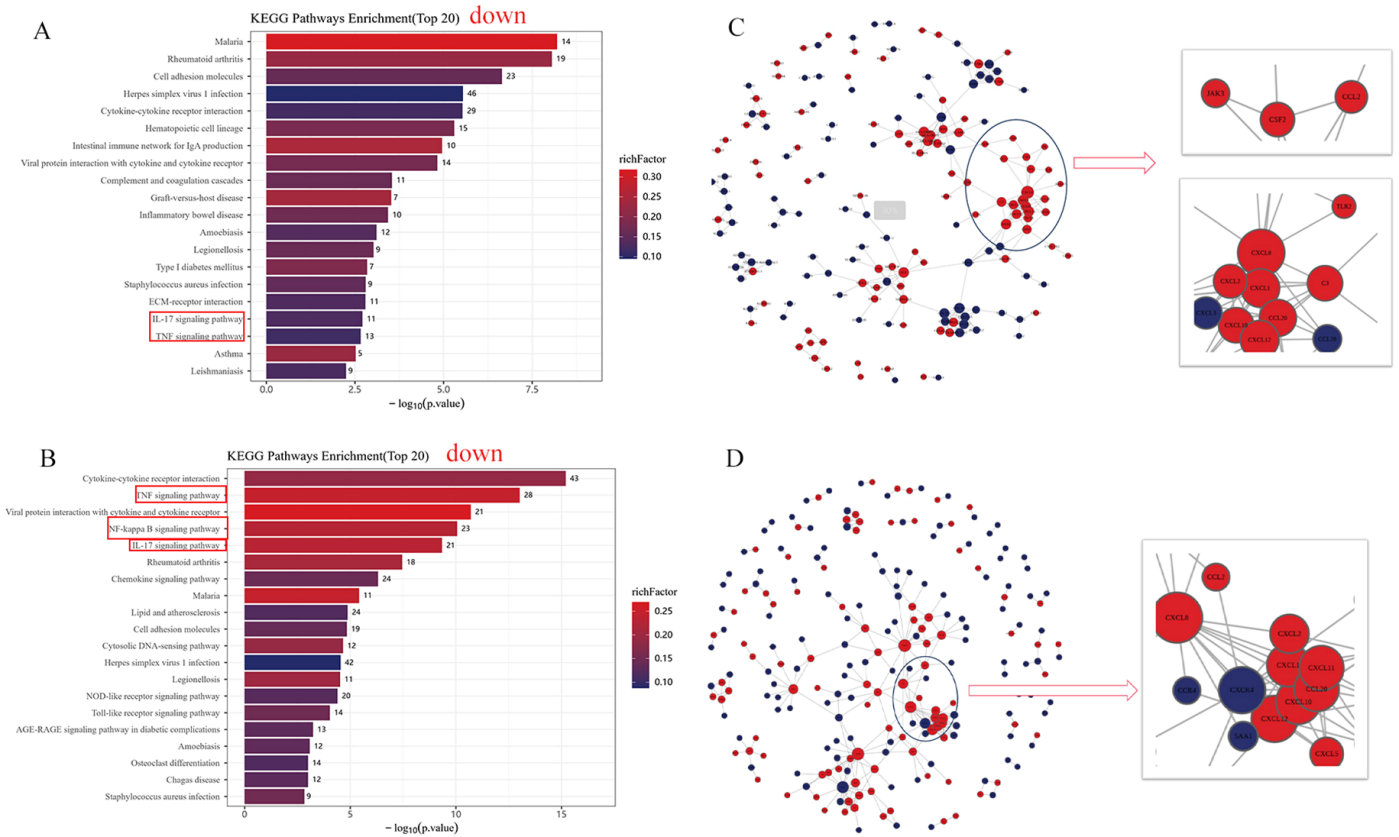
RNA sequencing analysis of lipopolysaccharide (LPS)–treated shCMTM3-HUVECs. **(A)** Top 20 KEGG pathways representing DEGs between shCMTM3-HUVECs and shsham- human umbilical vein endothelial cells (HUVECs). **(B)** Top 20 KEGG pathways between LPS-treated shCMTM3-HUVECs and LPS-treated shsham-HUVECs. **(C)** PPI network analysis of DEGs between shCMTM3-HUVECs and shsham-HUVECs. **(D)** PPI network analysis of DEGs between LPS-treated shCMTM3-HUVECs and LPS-treated shsham -HUVECs.

To further determine the role of CMTM3 in the lung endothelial cells during the development of ARDS, we treated shCMTM3-HUVECs and the shsham-HUVECs with LPS for 6h. Subsequently, we harvested total cellular RNA and performed sequencing and RNA transcriptome analysis. Differentially regulated genes (DEGs) were identified between the LPS-treated shCMTM3-HUVECs and shsham-HUVECs. Functional enrichment analysis was performed to determine the upregulated or downregulated KEGG pathways enriched by the DEGs. Our data showed that the TNF signaling pathway and NF-κ B signaling pathway were downregulated in the shCMTM3-HUVECs compared to the shsham group (**Figure 8A**). Furthermore, compared to the LPS-stimulated shsham-HUVECs, LPS-stimulated shCMTM3-HUVECs showed downregulation of the IL-17 signaling pathway, TNF signaling pathway, and NF-κ B signaling pathway (**Figure 8B**).

PPI analysis also demonstrated upregulation of VE-calmodulin-dependent factors such as CCL2, CXCL8, and CXCL10 in the shCMTM3-HUVECs compared to the shsham group (**Figure 8C**). Furthermore, expression levels of CCL2, CXCL8, and CXCL10 were further increased after LPS stimulation in the CMTM3ko-HUVECs (**Figure 8D**).

### 3.7 Effect of CMTM3 knockdown on the survival outcomes after ARDS modeling

**Figure 9:**
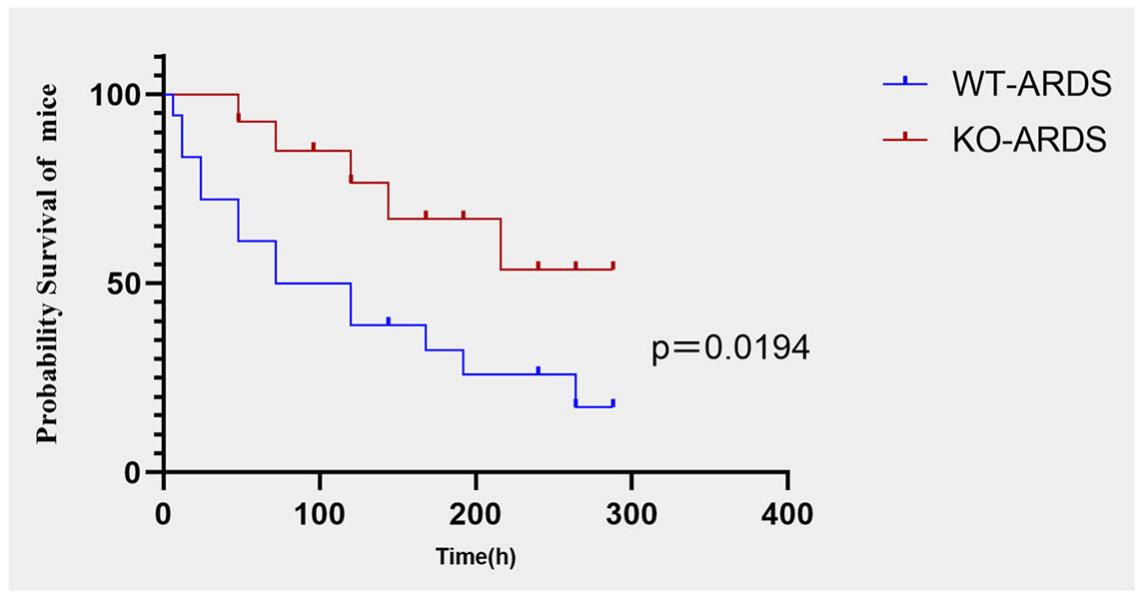
Kaplan–Meier survival curve analysis shows survival rates of the CMTM3ko-ARDS and WT-ARDS mice (*n* = 15 per group).

Finally, we analyzed the effects of CMTM3 knockdown on the survival outcomes of the ARDS model mice. Kaplan–Meier survival curve analysis demonstrated that the survival rate of the CMTM3ko-ARDS model mice was significantly longer than the WT-ARDS model mice (*p* = 0.0194, **Figure 9**).

## 4 Discussion

The pathogenesis of ARDS has not been fully elucidated. In ARDS, systemic inflammatory response triggered by the inflammatory cells leads to the release of excessive amounts of pro-inflammatory mediators and cytokines, which in-turn damages the alveolar-capillary barrier leading to lung injury (18). The alveolar-capillary barrier consists of thin layers of alveolar epithelial cells and capillary endothelial cells separated only by a thin basement membrane to facilitate gas exchange (19). Inflammatory response during ARDS results in the secretion of pro-inflammatory factors, such as TNF-α and IL-6 (20), which promote recruitment of inflammatory cells from the capillaries to the intra- and inter-alveolar compartments. The inflammatory cells produce large amounts of proteolytic enzymes, which extensively damage the alveolar endothelial and epithelial cells. This significantly reduces the levels of alveolar surface-active substances and leads to an increase in permeability and shedding of endothelial surface anticoagulant molecules, including coagulation regulatory proteins and the endothelial cell protein C receptor. Furthermore, upregulation of pro-coagulant molecules contributes to the formation of thrombus in the microvessels and leakage of the plasma fluids into the alveoli and the interstitium. Subsequently, this results in pulmonary edema and formation of hyaline membranes inside the alveoli and atelectasis (21). Throughout the injury process, pulmonary vascular endothelial cells are both damaged as well as activated by various factors. This alters their metabolic and regulatory functions and contributes further to the development and progression of ARDS (22). Damage to the pulmonary endothelial cells impairs endothelial cell-to-cell connectivity and increases vascular endothelial cell permeability, thereby leading to endothelial barrier dysfunction and disease progression (23). Therefore, early identification and intervention of pulmonary endothelial cell damage is necessary to suppress endothelial cell dysfunction and improve the prognosis of ARDS patients.

Previous studies have shown that CMTM family members regulate tumor cell growth and migration in different types of cancer (24). Yuan et al. demonstrated that increased CMTM3 expression in gastric cancer cells inhibited tumor cell growth and promoted apoptosis (12). However, the function of CMTM3 in normal cell types has not been studied in detail.

Our study showed that CMTM3 expression was significantly increased in the HUVECs that were treated with LPS or hypoxia/reoxygenation. The highest CMTM3 expression was observed in HUVECs stimulated with LPS for 6h (*p* < 0.001) and 4h of hypoxia followed by 2h of reoxygenation (*p* < 0.001). Furthermore, in the *in vitro* LPS-ARDS modeling experiments, CMTM3-overexpressing HUVECs showed significantly higher permeability (*p* = 0.0017), whereas CMTM3 knockdown HUVECs demonstrated reduced permeability (*p* = 0.0003). Moreover, in the *in-vitro* hypoxia/reoxygenation-ARDS model experiments, CMTM3-overexpressing HUVECs showed increased permeability (*p* = 0.002), whereas CMTM3 knockdown HUVECs showed reduced permeability (*p* < 0.0001).

We also successfully generated CMTM3 knockout mice and induced ARDS using the tracheal drip HCL method or tracheal drip LPS method. After 24h of HCL-ARDS modeling, the EB leakage in the lungs of the CMTM3ko mice was significantly attenuated compared to the WT mice (*p* < 0.0001). Lung injury was more severe in the tracheal drip HCL model compared with the tracheal drip LPS model. However, in the LPS-ARDS model, lung leakage was significantly reduced in the CMTM3ko mice compared to the WT mice (*p* < 0.0001). Chrifi et al. reported that Consistent with the increased monolayer cell permeability of HUVECs after overexpression of CMTM3-overexpressing HUVECs enhanced permeability and endothelial barrier function as shown by transendothelial electrical impedance measurements *in vitro* (15). This suggested that CMTM3 played a key mechanistic role in the endothelial cell dysfunction underlying ARDS by affecting endothelial cell permeability. RNA sequencing data analysis also showed that the expression of CCL2, CXCL8, and CXCL10 was upregulated in the shCMTM3 HUVECs. Furthermore, the expression of CCL2, CXCL8, and CXCL10 was significantly increased in the shCMTM3-HUVECs compared with the shsham group after LPS stimulation. Previous studies have demonstrated that increased expression of CCL2, CXCL8, and CXCL10 is dependent on the expression of VE-Cadherin (25). Maintenance of vascular endothelial integrity is dependent on the vascular endothelial intercellular junctional structures. Vascular endothelial cadherin (VE-cadherin) is located throughout the basement membrane and forms cis-dimers on the cell surface. The homeostatic level of VE-cadherin is dependent on the rate of endocytosis and degradation (26). In the pathological state, VE-cadherin on the cell membrane surface undergoes phosphorylation after stimulation by factors such as VEGF and LPS, and dissociates from the intercellular junctional complex. Subsequently, VE-cadherin is endocytosed by the vesicles and incorporated into the early endosomes. This results in the disruption and increased permeability of the vascular barrier (27). We found that the expression levels of VE-cadherin–dependent genes such as CCL2, CXCL8, and CXCL10 were upregulated in the LPS-stimulated shCMTM3 HUVECs compared with the controls. This suggested that the knockdown of CMTM3 exerted a protective effect against vascular endothelial damage in the LSP-stimulated HUVECs. Therefore, we hypothesize that CMTM3 modulates endothelial cell permeability by affecting endothelial intercellular junctional structure and function via VE-cadherin. CXCL8 (chemokine IL-8) is a pro-inflammatory cytokine with the ability to chemotactically attract neutrophils to sites of inflammation and inhibit neutrophil apoptosis. IL-8 is involved in ARDS mainly through the chemokine receptor CXCR2, which regulates neutrophil migration (28). Furthermore, IL-8 promotes angiogenesis (29). In our study, RNA sequencing analysis showed that the expression levels of IL-8 were elevated in the CMTM3 knockdown HUVECs. However, we did not observe upregulation of the IL-8 pro-inflammatory pathway and the expression of CXCR1/CXCR2, a related receptor. This suggested that upregulation of IL-8 in the CMTM3 knockdown HUVECs may promote angiogenesis, but the specific mechanism of action needs to be explored in further studies.

Inflammatory response is an important mechanism underlying the pathogenesis of ARDS. In the preliminary stages of ARDS, cells of the intrinsic immune system, such as macrophages, neutrophils, and nonspecific T cells, as well as other immune response-related proteins act as first-line effectors to scavenge antigens and induce an inflammatory response at the site of injury. This is followed by effective control of the inflammatory response by specific immune cells such as T cells and B cells (30). TNF-α and IL-6 play an important role as pro-inflammatory factors in the development of ARDS. TNF-α is an early response cytokine that plays a critical role in triggering inflammatory response. Therefore, anti–TNF-α drugs are the earliest interventions to enter clinical trials for ARDS. Toll-like receptor 4 (TLR4)–NF-κB signaling pathway, NF-κB signaling pathway, and p38 mitogen-activated protein kinase (p38MAPK) signaling pathway are the main inflammation-signaling pathways that are associated with the development of ARDS (26, 27, 31). LPS stimulation of HUVECs activates the downstream NF-κB signaling pathway by recognizing and binding transduction signaling receptors on the cell membrane. Furthermore, other inflammatory factors, such as TNF-α can directly act on the upstream protein kinase IKK through one or more signaling pathways. The phosphorylation of NF-κB inhibitory protein (IκB) promotes dissociation of the active NF-κB, which then translocates from the cytoplasm into the nucleus and regulates expression of several inflammation-related genes. In addition, positive feedback activation of NF-κB leads to a dramatic increase in the recruitment of pro-inflammatory and chemokine-mediated inflammatory cells, thereby significantly enhancing the inflammation response (31). In this study, expression levels of inflammatory factors such as IL-6 and TNF-α were significantly elevated in the adCMTM3 HUVECs (*p* = 0.0004 and *p* = 0.0072) and significantly reduced in the shCMTM3 HUVECs (*p* < 0.0001 and *p* = 0.0174) compared with the controls after LPS stimulation. Similarly, expression levels of IL-6 and TNF-α were significantly increased in the adCMTM3 HUVECs (*p* = 0.0078 and *p* = 0.0068) and significantly reduced in the shCMTM3 HUVECs (*p* < 0.0001 and *p* = 0.0006) compared to the controls after hypoxic/reoxygenation stimulation. In the experiments with mice, results from both the tracheal drip HCL and LPS models showed that lung injury was significantly reduced in the CMTM3ko-ARDS mice compared to the WT-ARDS mice. Furthermore, IL-6 and TNF-α levels were also reduced in the lungs of the CMTM3ko-ARDS mice compared to the WT-ARDS mice (*p* < 0.05) in both the tracheal drip HCL and LPS models. This suggested that CMTM3 knockdown decreased the production of pro-inflammatory factors IL-6 and TNF in the vascular endothelial cells after LPS and hypoxic stimulation, thereby downregulating inflammation in the ARDS mice. Therefore, we performed RNA sequencing analysis of the shsham and shCMTM3 HUVEC cells and observed that the IL-17 signaling pathway, TNF signaling pathway, and NF-κ B signaling pathway were significantly reduced in the shCMTM3 HUVECs compared with the shsham-HUVECs when stimulated by exogenous LPS. This result suggested that CMTM3 knockdown effectively inhibited LPS-induced inflammation in the endothelial cells inflammation. Taken together, this suggested that CMTM3 knockdown may effectively reduce lung damage by inhibiting inflammation and related pathways during the onset of ARDS.

Most importantly, in our *in-vivo* mouse ARDS modeling experiments, the survival rate of the CMTM3ko-ARDS model mice using the tracheal drip LPS method was significantly higher than that of WT ARDS mice (*p* = 0.0194). This suggested that the knockout of the CMTM3 gene not only ameliorated lung injury and inflammation in the ARDS model mice, but also improved the survival outcomes of the mice.

This study has a few limitations. First, we used HUVEC cells instead of lung endothelial cells (HPMECs) as our *in-vitro* model. Although both are endothelial cells, there may be few functional differences between them. Therefore, we plan to follow up by performing similar experiments with the HPMECs regarding the mechanistic action of CMTM3. Second, we used only male mice for the murine ARDS model. Therefore, in the future, to avoid gender bias, we will analyze the function of CMTM3 in female mice through ARDS modeling, and compare data with the male mice. Thirdly, our results suggested that CMTM3 regulated endothelial cell permeability, but the exact molecular mechanisms are unknown. Therefore, we will follow up with co-localization and CO-IP experiments to further investigate the mechanistic role of CMTM3 in ARDS.

In summary, our study suggested that CMTM3 promoted vascular endothelial permeability dysfunction and facilitated inflammation in ARDS. However, knockdown of the CMTM3 gene can effectively protected against vascular endothelial dysfunction and inflammatory lung injury caused by ARDS and improved the survival outcomes in the ARDS mice. Therefore, our study suggested that CMTM3 is novel therapeutic target for ARDS.

## Data Availability

The original contributions presented in the study are included in the article/supplementary material. Further inquiries can be directed to the corresponding author.
